# Supplementary material for: Preliminary assessment of three quantitative approaches for estimating time-since-deposition from autofluorescence and morphological profiles of cell populations from forensic biological samples
Source: PLoS One. 2023 Oct 12;18(10):e0292789. doi: 10.1371/journal.pone.0292789 (PMC10569564; doi:10.1371/journal.pone.0292789)
Supplement: S3 Table — MSE ln(days): Mean squared error in ln(days). MSE days: Mean squared error in days. (DOCX) [file pone.0292789.s003.docx]

**Supplemental Table S3: Mean squared prediction error in the N-fold cross-validation set.**

|  | **MSE ln(days)** | **MSE days** |
| --- | --- | --- |
| LASSO | 1.67 | 1.2E+10 |
| RIDGE | 1.77 | 2.7E+8 |
| GBM | 1.28 | 6995.2 |
| GLMM | 1.41 | 9561.9 |

Legend:

MSE ln(days): Mean squared error in ln(days)

MSE days: Mean squared error in days
